# Clinical Handover as Craft, Not Checklist

**DOI:** 10.1111/1742-6723.70247

**Published:** 2026-04-06

**Authors:** Aloha Hufana Ambe, Nathan Brown, Daniel Bodnar, Tobias Grundgeiger, Sean Rothwell, Ben Matthews

**Affiliations:** ^1^ Electrical Engineering and Computer Science The University of Queensland Australia; ^2^ Emergency and Trauma Centre Royal Brisbane and Women's Hospital Australia; ^3^ Institute Human‐Computer‐Media Julius‐Maximilians‐Universität Würzburg Germany

**Keywords:** clinical handover, clinical judgement, handover as craft, patient safety, rethinking quality and safety

## Abstract

Handover is one of the most studied elements of clinical care and its importance to the good continuity of care and patient outcomes is well‐accepted. Unfortunately, efforts to improve the quality of clinical handovers have led to an environment of compliance exemplified by mnemonics, templates and audits that assume or suggest handovers to be stable, reproducible processes that can be judged by conformity to checklists. However, handovers are highly heterogenous, personalised and nuanced events that do not easily fit into tightly structured models. We draw on observational work in a 2‐year, ongoing collaboration between a large emergency department and a human‐centred design research team, and argue that handover is essentially a craft: a skilled practice shaped by timing, uncertainty, recipient needs and jurisdictional boundaries. We suggest that standardisation of handovers can work against quality, and that simple checklists threaten to flatten rather than enhance their true craft or value. Shifting how clinical handover is understood, taught, assessed and supported, by emphasising the importance of craft over completeness may be the secret to ensuring quality.

## 1 Why This Matters Now

Handover is often treated as both the solution and the culprit: a place where safety is protected, and a place where failure is blamed. EMA has long documented that handover deficiencies can have adverse effects, and that efforts to improve handover meet real constraints at the bedside and in context [1, 2, 3]. Given these stakes and constraints, it is not enough to treat handover as a compliance problem, or a straightforward test of competence.

At the same time, much of the handover conversation still assumes that standardisation is the desirable endpoint. Structure helps, especially early on, but in day‐to‐day ED work handover is shaped by timing, uncertainty, workload and who is receiving. That is why it cannot be reduced to a stable script. It is a process, yes, but it also has the qualities of a craft.

We use *craft* deliberately. Clinicians bring clinical knowledge, pathways, rules and a deep understanding of risk. But handover also requires the subtle work of making things happen in real conditions: adapting the patient story for the recipient, deciding what matters now and negotiating boundaries. That work is skilled, often invisible and it is frequently what makes the transfer safe.

## 2 What Our Work Shows, in Brief

This Opinion draws on fieldwork that has largely been published outside emergency medicine venues [4, 5, 6]. Over 2 years, we worked closely with ED clinicians to understand how coordination is accomplished in practice. Our studies focused on sites where handover and referral work become especially visible: shift and team handover [6], referral calls out of the ED where responsibility is negotiated across jurisdictional seams [4], and a participatory workshop that surfaced what matters to clinicians when technological change is proposed [5]. We also examined ambulance‐to‐ED handover across triage and bedside (under review).

Across our studies, one finding kept reappearing. The patient story is not simply ‘transferred’—it is shaped for purpose. What is relevant depends on the recipient, circumstances and the next decision that needs to be made. Ambulance‐to‐triage, ambulance‐to‐bedside and referral calls each demand different forms of case description. Across these handover and referral moments, expertise gradients matter. What an experienced clinician can infer, compress, or flag differs from what a novice can safely manage; what a receiver needs depends on what they already know and what they are accountable for next; effective handovers depend on interactions being tailored to each party's expertise, accountabilities and scope for action. Handovers are not one‐directional information transmission—they are interactions where mutual understanding must be an achievement shared by both parties.

This resonates with handover sometimes being ‘lost in translation’ between paramedics and ED receiving staff, where shared understanding can be hard to achieve [2], and with bedside handover being limited by practical constraints and staff acceptance rather than instruction alone [1]. Our argument is to make the craft more explicit, so we can talk about handover without defaulting to blame.

## 3 The Risk of Treating Craft as Checklist

Checklists and mnemonics have their place. They can provide a shared starting point, especially for novices and education sessions can improve some aspects of shift handover or provide useful checks for completeness [7]. Our point is not that structure is bad, but that structure on its own cannot carry the full weight of what handover needs to achieve in practice.

The problem starts when the scaffold becomes the gold standard. When handover is judged primarily by checklist completion, we risk misrecognising competence. A clinician can ‘tick the boxes’ and still fail to achieve shared understanding. Another clinician can omit a category yet communicate the case in a way that is safer and more actionable for that moment. No two handovers are the same, because the patient, the receiving team, the time pressures and the surrounding workload are never the same. A single rubric struggles to hold that variability.

This is also where accountability can become skewed. If a handover is implicated in an adverse event, it is tempting to look for missing items and treat those as the cause. But handover is often only the visible surface of a chain of coordination work. If we focus only on the moment, we may miss what made it hard and we may end up negatively judging adaptive work instead of improving the system around it.

## 4 Making Work Visible, With Care

Lucy Suchman, an anthropologist of technology and work, reminds us that making work visible is never neutral: visibility changes how work is valued, managed and judged [8]. The representations that travel (templates, forms and summaries) can stand in for working knowledge and end up being used for purposes far from the worksite, including audit and performance evaluation [8]. In emergency care, this matters because the visible artefact is often not the work itself, but only a thin representation of it.

If what travels is only a simplified account, it can stand in for working knowledge and shape how performance is judged, rather than how work is supported [8]. A form can become ‘the handover’. A template can become ‘the standard’. In that framing, the craft gets flattened. In practice, skilled craftwork involves artful adaptation, reconciliation and coordination.

To improve handover in a meaningful way, we need to be explicit about preservation of the craft. Preservation does not mean resisting structure or technology. It means refusing to let what is easiest to document become a substitute for what is safest to do.

## 5 Where This Thinking Should Take Us

We need to shift what we treat as the problem. Too often, handover improvement starts and ends with standardising content. That helps people begin, but it does not account for how handover works in real conditions. Tools that flatten skilled practice invite the possibility of audit by non‐experts, who can easily check for ‘compliance’ without recognising the experienced clinical judgement in action, or appreciating the broader context of the work.

*Training*: If we are going to call handover critical, then we must teach it as a clinical craft, not as a short module on a mnemonic. Early structure helps, but competence develops through experience, exposure to variation, guided reflection and feedback from experienced clinicians. Education interventions can contribute [7], but the content needs to match the work: tailoring the story to the recipient, signalling uncertainty, negotiating responsibility and achieving shared understanding under pressure [4, 6]. The challenge is to embed the notion of craft as a foundational element in the education of clinical handover.

*Assessment and audit*: Compliance checking will always exist but if it is the sole metric of quality, then it can become a problem. Review processes need to account for context, workload, purpose and expertise gradients and avoid turning handover into a single performance moment detached from the coordination that surrounds it [1, 2, 3, 8]. When handovers are investigated, they should not be treated as a discrete failure point explained by ‘missing’ content. The key question is what the handover was trying to achieve under the constraints of that moment. This is not about excusing poor practice. It is about holistically seeing enough of the work to improve the system, not just a narrow practice. Otherwise, we risk judging only a thin slice of work, rather than acknowledging the invisible coordination that made the handover possible, or difficult, in the first place [8]. The challenge is, if not compliance by checklist, then how can we best assess the quality and safety of handovers? In other serious crafts, experience and expertise are necessary to evaluate performance, rather than conformity to a template or standard.

*Technology*: EDs will continue to be offered tools to ‘fix’ or ‘improve’ handover, increasingly including AI‐enabled documentation and decision support. The goal should not be to reify handover as a uniform process, but to support the craft: preserving what matters, supporting coordination across boundaries and avoiding unintended burdens [4, 5, 6]. Doing this well requires meaningful engagement with staff to design/adapt appropriate tools [9]. With that in mind, the challenge will be, for any new systems or AI tools, to design and implement them so as to preserve the craft of handover, rather than flattening it into what is easier to standardise or measure.

## 6 Closing

Treating handover as a checklist‐able process gives clean categories, but risks losing the craft clinicians rely on to keep care safe across uncertainty and pressure. If we take that craft seriously, then we should not be satisfied with teaching handover as a short mnemonic, or judging it as a single auditable moment. We should train for judgement and tailoring, assess quality in context rather than equating it with compliance, and approach accountability with an eye to the invisible coordination work that sits behind what a template can show [8]. And when new digital and AI‐enabled systems arrive promising greater completeness and standardisation, we should judge them by a higher bar: not whether they produce cleaner records, but whether they help clinicians keep care safe and moving, without eroding the craft that makes handover work.

## Funding

This research was supported by the Australian Research Council (DP220102349, Traversing Jurisdictional Seams: Information Support Needs for the Trauma Patient Pathway).

## Ethics Statement

This Opinion draws on studies approved by the University Human Research Ethics Committee (Ref: 2019/HE002939) and the participating health service Human Research Ethics Committee (PR/2022/QRBW/59202). No new data were collected for this article.

## Conflicts of Interest

The authors declare no conflicts of interest.

## Data Availability

Data sharing not applicable to this article as no datasets were generated or analysed during the current study.
